# A comprehensive review of types, synthesis strategies, advanced designing and applications of aerogels

**DOI:** 10.1098/rsos.241975

**Published:** 2025-05-21

**Authors:** Endazenaw Bizuneh Chemere, Thobile L. Mhlabeni, Washington Mhike, Mapula Lucey Mavhungu, Mxolisi Brendon Shongwe

**Affiliations:** ^1^ Department of Chemical, Metallurgical and Materials Engineering, Tshwane University of Technology, Pretoria, South Africa

**Keywords:** aerogels, synthesis methods, advanced designs, interaction mechanisms, pollutants

## Abstract

Aerogels are gaining interest from researchers for various applications in different disciplines due to their unique characteristics, such as high porosity, low density and vast surface area. Because of their outstanding physical and chemical properties with adjustable surface chemistry, aerogels have various applications, including supercapacitors, drug delivery, thermal insulation, etc. Due to their high porosity and surface area, aerogels are applicable for the removal of organic and inorganic pollutants, which could have detrimental impacts on both human health and the environment. Numerous reviews of aerogel preparations and applications have been published. However, there has not been an organized approach to aerogel synthesis methods, types and applications with the mechanisms in their respective applications. The aerogels' pollution removal methods and composite fabrications are thoroughly described. A summary of the diverse uses and exciting prospects of aerogels in environmental pollution control is provided. Additionally, how contaminants and porous nanoparticles interact are examined. More significantly, novel designs are suggested for the production of advanced aerogels. The mechanisms of the porous materials in their applications are also meaningfully guided by this review, which helps to design and produce advanced porous materials.

## 1. Introduction

Aerogels are light in weight, highly porous materials with an extremely high surface area formed by a gelation process in which the liquid part of the gel is removed and substituted with gas without collapsing the gel**,** resulting in no size change [1,2]. They can be prepared from organic, inorganic and hybrid molecular precursors which are normally by a sol–gel process and a special drying technique with which the three-dimensional structure and the highly porous network are conserved. Their outstanding physical and chemical properties, such as low density, high surface area, high porosity and adjustable surface chemistry, render them as suitable adsorption media that remove a variety of environmental and human health pollutants [3]. The rapid development of industrialization and human population remains challenging for pollution control and remediation [4]. According to the Lancet Commission on Pollution and Health [5], the attention towards pollution and its harmful effects on the environment and human health has been neglected for decades by governments and the international development community. The issue is particularly severe in developing nations because of overcrowding, uncontrolled urbanization and the growth of industry. Poor air quality results from this, particularly in nations with social inequalities and lack of knowledge about environmentally friendly practices. The use of fuels such as wood fuel or solid fuel for domestic needs due to low incomes exposes people to bad-quality, polluted air at home. Both natural and anthropogenic (such as industrialization, urbanization and globalization) activities are all drivers of pollution [5].

To remove or break down pollutants, various methods and technologies, including reverse osmosis, ozonation, biological treatments, nanofiltration, advanced oxidation processes and engineered biodegradation/biofiltration have been employed [6]. However, these technologies have important disadvantages, such as high energy consumption, membrane fouling, the challenge of brine disposal, the need to remineralize the permeate and insufficient removal of nascent contaminants [7,8]. Moreover, because of their expensive capital and maintenance, these technologies come with the risk of being novel and are not contained by other nations quickly. When taken as a whole, these disadvantages necessitate more affordable and versatile witness materials [9].

Currently, aerogels have been attracting researchers’ attention for environmental remediation of their unique features such as low density, tunable porosity, high surface area and easy functionalization [9,10]. As it has been already stated earlier, because of their outstanding physical and chemical properties with adjustable surface chemistry, aerogels have various applications. They are described as such innovative materials for the adsorption of a wide range of water pollutants due to their porous micro- or nano-structures [9]. Aerogels are fundamentally low-cost, safe, sustainable and highly versatile materials that may be recycled [7]. Drying a gel under supercritical conditions led to replacement of the liquid by air which makes it a light, porous and yet solid-phase substance that demonstrates a variety of remarkable properties suitable for various applications such as water treatment [11], air purification [12,13], biomedical devices [14], catalysts [15], drug delivery [16], food technology [17], thermal insulation [18], chemical sensor [19], energy storage [20], acoustic transducer [21], waterproof coatings [22], wearable fabrics [23] and smart devices [24] to mention just a few.

Nowadays, the modern world faces significant challenges in dealing with water and air pollution because of the fast growth of heavy industry and climate deterioration [25]. The effects of some pollutants, such as heavy metal ions [26,27], organic dyes [26,28], carbon dioxide [29], nitrogen oxides (NO_x_) [30] and volatile organic compounds (VOCs), on the environment have come under close examination in recent years. These pollutants may be poisonous or cancer-causing, which could have negative consequences on the environment as well as human health. In comparison with conventional adsorbents, nanostructures, etc., aerogels prepared from organic, inorganic and hybrid materials showed fascinating performance in the removal of pollutants, such as heavy metal ions, dyes, polycyclic aromatic hydrocarbons, oils and organic solvents [31]. Prior to discussing aerogels and their uses, it’s critical to roughly highlight pollution and contaminants.

## 2. Pollution and pollutants

### 2.1. Pollution

The introduction of possibly hazardous elements or materials into the environment brings about environmental pollution. Almost all natural and human activities that result in the degradation or deterioration of the quality of the natural environment are considered pollution [32,33]. These materials negatively impact human health and damage the environment. Pollution can happen when pollutants, such as solids, liquids, gases or a form of energy such as heat, sound or radioactivity introduced into the environment at a rate faster than they can be decomposed, dispersed, recycled or stored in some harmless form. Although pollution has been known for many years, it continues to be the biggest issue facing humanity and the primary cause of disease morbidity and mortality [32,34,35]. Pollution of the environment can occur both by natural phenomena including volcanic eruption and wildfire and by anthropogenic activities such as industrialization, agriculture and transportation [34]. Pollution is transboundary and is not limited to the environment of industrialization, urbanization, exploration and mining. Pollution is a global issue because pollutants can travel across borders via a variety of routes, primarily air and water [36,37].

### 2.2. Pollutants and their sources

Heavy metal ions [38], organic dyes [39,40], organic pollutants [41,42], greenhouse gases like CO_2_ and exhaust gases (NOx and Sox) [31] are examples of environmental pollutants.

#### 2.2.1. Heavy metal ions

Because of industrial processes and technological advancements, heavy metal ions are consistently being released into the environment, posing a serious risk to public health and the environment. Heavy metal ions, which are extremely harmful pollutants in water resources, cause global water pollution [43]. Different hazardous metals are released into the environment by the rubber industries, electroplating, mining, oil refineries, staining, stabilizers, batteries, metallurgy and electrical industries. The hazardous heavy metal ions include lead (Pb^2+^), mercury (Hg^2+^), cadmium (Cd^2+^), arsenic (As (III)), chromium (Cr (VI), etc [44].

#### 2.2.2. Dyes

The presence of organic pollutants in water resources poses a major threat to the environment, wildlife and human health. Industries ranging from small-scale tanneries to large-scale textile, food, cosmetic and pharmaceutical sectors use dyes. Primarily, textile industries release huge amounts of dyes into the ecosystem [45,46]. The dye’s complex structures, which are made up of aromatic rings bound to various functional groups with an electron, can absorb light in the 380−700 nm range. The presence of chromogens and chromophores gives them colour. Because azo groups emit amines and benzidines into the air, they are highly carcinogenic among both synthetic and natural dyes. In addition, the dyes have prolonged half-life in the environment which due to their non-biodegradable nature creates risks [45]. Dyes can be categorized as follows [31,47]:

*In terms of chemical structure*: Anthraquinone, azo, phthalocyanine, indigo, nitro, nitroso, etc.

*In terms of charge in aqueous medium*: cationic (all basic dyes), anionic (direct, acidic and reactive dyes) and non-ionic (dispersed dyes)

*In terms of chemical nature*: acidic, azoic, basic (cationic), direct, disperse, oxidation, reactive, vat, solvent, sulfur, etc.

#### 2.2.3. Oils and organic solvents

A steadily increasing amount of waste, such as oil-filled industrial effluents, and the leakage of organic solvents (benzene, toluene, cyclohexene and dichloromethane) also threaten public health and territorial ecosystems [48]. Oil becomes a pollutant of the environment when it reaches the surface of the earth, where it is not supposed to be. Crude oil derivatives are included in the category of oil pollutants for the same reason [49]. Oil spills from industry, including those involving gasoline, olive oil, pump oil and crude oil, as well as organic solvent leakages such as nitrobenzene, tetrahydrofuran (THF) and chloroform, have had disastrous consequences for aquatic and marine environments. Globally, an estimated 1.2 million tons of oil are released into the environment each year, both on land and in water [50]. As a result, there has been increased focus globally on the collection and removal of oil and organic contaminants from water and wastewater [31].

#### 2.2.4. Polycyclic aromatic hydrocarbons (PAHs)

Polycyclic aromatic hydrocarbons (PAHs) have been garnering substantial attention because they have a long history of being teratogenic, mutagenic and carcinogenic. Moreover, PAHs do not undergo rapid degradation under natural conditions and require external remediation. According to the Environmental Protection Agency of the US, 16 PAHs such as naphthalene (Nap), acenaphthylene (Any), acenaphthene (Acp), benzo (a) pyrene (Bap), fluorine (Fl), etc., have been categorized as priority pollutants that require stringent regulation [51]. The burning of coal, biomass, fossil fuels, automobile emissions, petrogenic activity and volcanic eruptions are some sources of PAHs. Through volatilization, surface runoff and other processes, PAHs generated from these sources partially leach into the air and water systems from the soil leading to pollution [52].

## 3. Types of aerogels

**Figure 1.**
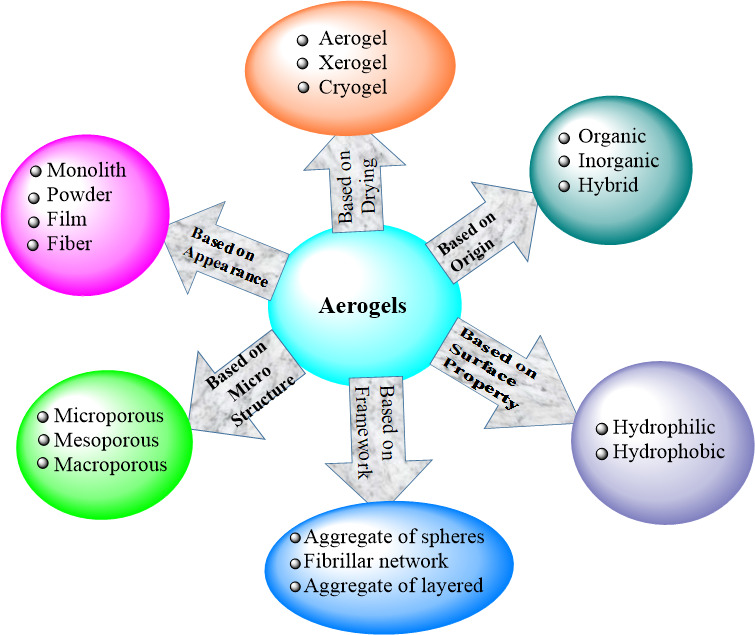
Classification of aerogels on different bases.

As shown in figure 1, on different bases, aerogels can be classified into different types. For example, based on the precursors (origin) used for the synthesis process, aerogels can be categorized as organic, inorganic and hybrid. Depending on the preparation methods (hydrogel drying method), aerogels can also be classified as aerogel, xerogel and cryogen. They can be classified as monolith, powder, film and fibre depending on their appearance [53]. Based on their microstructure, aerogels can also be classified as microporous (<2 nm) and mesoporous, (2–50 nm) [54], and macroporous (>50 nm) [55].

### 3.1. Aerogels based on drying method

**Figure 2.**
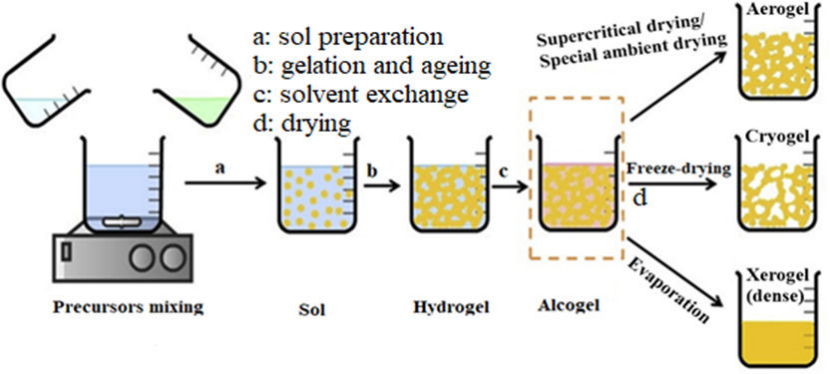
Schematic illustration of conventional aerogel synthesis process [55,56].

A schematic of the conventional aerogel production method and its parameters is displayed in figure 2. The sol-gel method is the most conventional and popular technique for synthesizing aerogel. It typically involves the following key steps: sol preparation, gelation, ageing and drying [56–58].

- The sol preparation step (a): When solid nanoscale particles form from a precursor material dispersed in a solvent, a colloidal suspension is created. This may involve hydrolysis and polycondensation reactions.
- Gelation and ageing (b): The gelation is the sol-to-gel transition step to form a hydrogel where the addition of an acid or base catalyst starts the polymerization process and creates an interconnected chain structure, causing cross-linking and branching particles. The ageing of the gel is to strengthen the gel’s backbone and mechanical strength; it is aged in its mother solution. There is also solvent exchange, which results in alcogel.
- Solvent exchange step (c): The solvent exchange procedure is done in steps because organic solvents and water have varying surface tensions, and a concentration gradient might cause pore collapse. The gel structure will be destroyed as a result of this collapse, and the substance will shrink. Gel samples are therefore moved from water to water and organic solvent solutions, with the organic solvent’s content gradually rising.
- The drying step (d): In this step, the solvent is extracted from the gel’s pores in a way that prevents gel fracture.

Among the above-mentioned steps, drying the gels is the key step that determines many properties of the aerogels. It is expected that the solvent, residues, byproducts and unreacted chemicals are removed, while the three-dimensional network is preserved intact. The three techniques that are most frequently employed to dry wet gels are supercritical drying, freeze-drying (lyophilization) and ambient (evaporation) [57,59]. Supercritical drying yields aerogel, freeze-drying (FD) yields cryogel and ambient drying (evaporation) yields xerogel [56]. Different physicochemical and morphological features can be produced by each drying process; however, all of them demonstrate ultralight weight and thermal insulation aerogels (e.g. silica aerogels) [60].

#### 3.1.1. Supercritical drying

Supercritical drying is the most popular method for producing lower-density aerogels. In the supercritical drying process, the solvent-rich gel, which is placed in a closed container, is exposed to a gas such as carbon dioxide (CO_2_) or methane. The procedure aims to increase the temperature and pressure inside the reactor over the critical points of the solvent which is found in the pores of the gel [61]. When the pressure and temperature in the reactor (autoclave) reach above a critical point, the surface tension is eliminated as the liquid becomes a supercritical fluid and every molecule of the fluid freely moves [62].

The supercritical drying methods can be high-temperature supercritical drying (HTSCD) or low-temperature supercritical drying (LTSCD) [61]. In HTSCD, by increasing the temperature and pressure inside the reactor (autoclave), the solvent is transformed into a supercritical fluid, and then gently vented at a constant temperature. In this drying technique, after the organic solvent is substituted for the hydrogel, the gel is inserted in a container for pressurizing and heating. When the supercritical state of the solvent is reached, the solvent discharges out from the gel. In LTSCD, the organic solvents such as methanol, ethanol and acetone in the gel can be substituted by CO_2_, which later will transform into supercritical CO_2_ (Sc-CO_2_). This transition, which finally addresses the development of gel, can only take place at a critical temperature that is close to the room’s temperature. One way to carry out this process is by pumping CO_2_ into the sample while flushing out the organic solvent and then removal of the ScCO_2_ at high pressure. Alternatively, you could flow the Sc-CO_2_ through the sample continuously to extract the organic solvent [63]. As has been mentioned earlier, the supercritical drying technique (using Sc-CO_2_ fluids) is the most widely used method because of its merits such as being easily available, non-toxic, chemically inert, mild operating conditions (temperature 31.1°C; and pressure 7.4 MPa) and non-combustible [64]. It is a complex and costly process and not suitable for large-scale production [65].

#### 3.1.2. Freeze-drying

The FD method in the aerogel synthesis is often considered as the best method for solvent removal to obtain dried products with the highest quality [66]. Freeze-dying is the widely used drying method in the aerogel preparation process which is usually a two-step process. The first is a pre-freezing step in which the wet gel is converted into a solid state at a temperature between −45 and −15°C. The second step is a sublimation step in which the liquid that is present in the solid state converts into gas under vacuum and it is a step that leads to the formation of pores in the aerogel matrix [67]. During the pre-freezing stage, the development of ice crystals from the liquid causes a volume increase, which causes the crust layers to shrink and fracture. Furthermore, the ice crystal grows at different rates, which causes the aerogel’s pore structure to be non-homogenous [68]. Water expands during the solidification of the process of FD, which frequently results in macroporous and cracks after sublimation [69]. In the synthesis of aerogels from amylomaize starch, aiming to produce porous materials, both supercritical-drying and FD methods were applied for the drying process, through which the former drying process produced porous aerogel with high specific surface area (197 m^2^/g) while the latter resulted in macroporous, with limited surface area (7.7 m^2^/g) [70].

#### 3.1.3. Ambient/air drying

Ambient or air drying is carried out at room temperature and pressure or in an oven set at a steady temperature until the weight remains constant. It is used in the drying of alcogel [71]. Solvent evaporation occurs directly during air drying, producing capillary tension ranging from 100 to 200 MPa. When the solvent is drained, creating a gel network, the pores compress as a result of the liquid-vapour meniscus receding within the pores [68]. Ambient drying has some merits such as low cost, low shrinkage, etc. By using both ambient and FD methods, a composite aerogel of fibre-reinforced polybenzoxazine demonstrated low shrinkage, self-extinguishing properties and flame retardancy [72]. A hybrid aerogel of cellulose acetate/polybenzoxazine was prepared by a low-cost ambient drying technique. The low-cost ambient drying aerogel demonstrated low drying shrinkage and superior mechanical properties under cryogenic conditions (−196°C), excellent thermal insulation and flame retardancy. With this ambient pressure drying approach, a 6.8% shrinkage rate could be controlled, mainly due to the enhanced skeleton by incorporating polybenzoxazine network chains [73]. Another study used the ambient pressure drying method and the sol–gel method to generate a polybenzoxamine composite aerogel with DOPO-HQ added as a flame retardant and the aerogel demonstrated excellent flame-retardant properties of phosphorus [72]. Despite being a simple procedure, ambient drying takes more than 48 h to complete [74]. Table 1 displays a summary of drying methods with their advantages and disadvantages.

**Table 1.** Brief description, advantages and disadvantages of drying methods.

drying method	description	advantage	disadvantage	reference
super critical drying	the scCO_2_ drying utilizes the high solubility of scCO_2_ to extract the solvent with no liquid-vapour interface, reserving the highly porous and textural structure of the liquid gel	low-temperature critical points, less energy-intensive, no liquid-vapour interface, highly porous and textural structure of the liquid gel	high production cost, non-eco-friendly because of organic volatile solvents for solvent exchange	[71,74]
FD	in the FD technique, the ice crystals in the frozen hydrogel are removed by sublimation at low pressure	low drying shrinkage, prepared materials are stable and efficient, low cost, applicable for drugs soluble in water or organic solvents	time consuming, results in smaller surface areas	[56,71,75]
ambient drying	the silica aerogel’s surface is hydrophobically altered during ambient pressure drying to remove the terminal silanol group (Si-OH), and the solvent is exchanged to a low-surface energy solvent like hexane to reduce shrinkage	easy technique, low drying shrinkage, low cost and low energy consumption	time consuming, non-eco-friendly friendly because of organic volatile solvents for solvent exchange	[72–75]

### 3.2. Aerogels based on their appearance

On the basis of appearance, aerogels are further classified into monoliths, powders, fibres and films.

#### 3.2.1. Monolithic aerogels

Monolithic aerogels are intriguing options for use in air purification due to their many pore structures, high specific surface areas, small sizes and other structural features. Furthermore, in real-world environmental cleanup applications, monolithic aerogels differ from aerogel powders and nanoparticles due to their distinct monolithic macrostructure [75]. Monolithic aerogels can be easily obtained by drying physical gels formed by linear uncross-linked polymers. Monolithic aerogels have excellent guest transport qualities and are appropriate for use in chemical separation, purification and storage as well as biomedicine. They also have robustness, durability and ease of handling and recycling [76]. They often have a uniform quality throughout and are homogenous in character. Their facile handling and shaping to required dimensions therefore open up a whole new range of applications in energy devices, building insulation, sound absorption and pollution adsorbents [53].

#### 3.2.2. Aerogel films

Various methods such as sol-gel, self-assembly, freeze casting and chemical vapour deposition (CVD) methods can be used to prepare aerogel films. Aerogel films are used for flexible electronics because of their unique properties such as high porosity, high specific surface area, light weight and tunable mechanical and electrical properties [77]. Aerogel films of organic, inorganic and organic–inorganic hybrid have been developed based on graphene [78,79], carbon nanotube [80,81], MXene [82], silica [83,84], cellulose [85,86], polyimide [87,88] and aramid nanofibre/ Mxene [82]. Aerogel films are showing promising performances in different applications. For example, a chitosan-derived composite aerogel film with the addition of melamine-phytic acid hybrids demonstrated high radioactive cooling and fireproof performances, with IR emissivity of up to 90.4% and solar reflectivity about 89.3% [89]. Aerogel films are also used in many other applications such as catalysis, [90,91], electronics [77], batteries [80], separation/filtration [77,92], thermal management [87,93] and piezoresistive sensors for real-time monitoring of subtle human activities such as carotid pulse beats, swallowing and facial expressions [94].

#### 3.2.3. Powdered aerogel

The development of new materials with complex structures and special strength, thermal and other attributes is the primary path for energy efficiency solutions in the building industry. With their great porosity and superior thermal insulation qualities, aerogels are appropriate materials [95]. Powdered aerogels are frequently used to improve the safety and insulating qualities of the materials. They are used as fillers in construction materials, electronic equipment and protective apparel such as fire jackets and spacesuits [96]. For example, in an experimental assessment aiming to evaluate the aerogel application as a parget powder component of concrete mixture to printing buildings, the thermal conductivity of aerogel-enhanced building decreased by 25% [95].

#### 3.2.4. Aerogel fibres

One-dimensional fibrous aerogel is a very flexible material that can be manufactured in a variety of ways, making it an outstanding material for textile thermal insulation [97]. At the present time, electrostatic spinning and wet spinning are the main techniques used to prepare and shape aerogel fibres. Since the electrostatic spinning offers a flexible and controllable process and makes use of a wide variety of spinnable materials, it is main moulding technique for aerogel fibres [98,99]. The main disadvantages of the electronic spinning technique are its high production cost, complex process requirements and the fact that it is mainly used in the production of nano-sized aerogel fibres. On the other hand, wet spinning is another essential technique for fabricating aerogel fibres as it provides a more straightforward and economical way to enable continuous extrusion [100]. Aerogel fibres are used in fireproof materials for thermal insulation [101], for smart and functional wearable devices [102,103], supercapacitors [104] and electromagnetic interference shielding [105].

### 3.3. Aerogels based on microstructure

**Figure 3.**
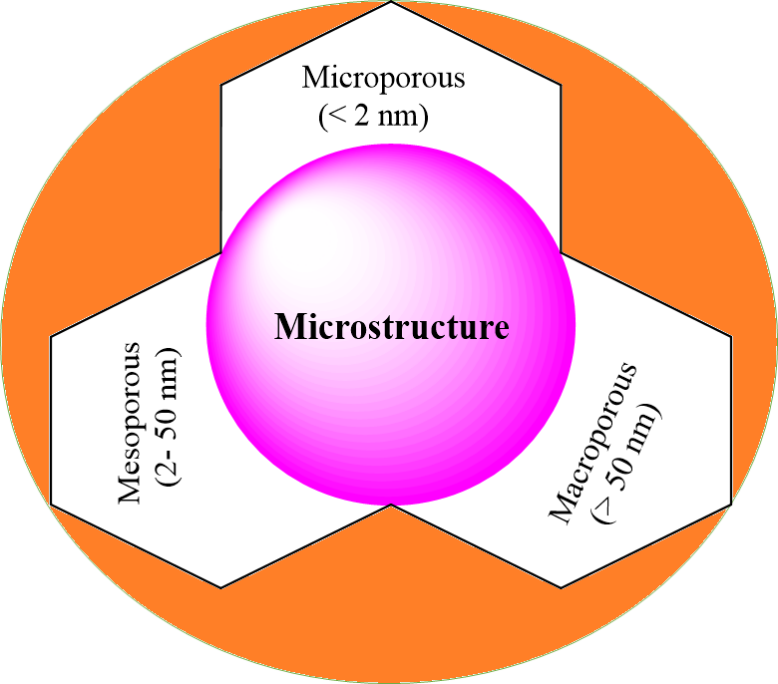
Aerogel classification based on microstructure.

Figure 3 illustrates the classification of aerogels based on microstructure. The terms ‘microporous’, ‘mesoporous’ and ‘macroporous’ refer to materials that have pore sizes of micro (<2 nm), meso (2–50 nm) or macropores (>50 nm), respectively [55].

#### 3.3.1. Microporous aerogels

Microporous aerogels can be prepared through the wet chemical approach, mainly by the sol–gel technique and an appropriate drying technique. These materials have fascinating physical properties such as ultra-low density, high specific surface area and adjustable surface chemistry [106]. The controlled microstructure renders them very useful materials for various advanced technologies such as thermal insulation [93,107,108], CO_2_ capture [109–111], catalysis [112], pollutant mitigation [93], sound absorption [113] and energy storage devices (e.g. supercapacitors) [114]. These materials also have attractive applications in food sciences. For example, a gelatin-based aerogel smart label using betacyanin as a response signal for detection of food spoilage was designed. The aerogel smart label produced a visually noticeable colour change in the presence of ammonia and displayed an instantaneous colorimetric response for food spoilage of shrimp. Additionally, the findings showed that the smart aerogel labels have a higher response sensitivity to volatile ammonia than the smart film labels [115].

#### 3.3.2. Mesoporous aerogels

These are porous materials having pore sizes ranging from 2 to 50 nm and they are distinguished by their easy controllability and homogeneity of pore size [53]. Mesoporous aerogels are prepared by wet chemical methods, mainly using the sol–gel technique [116–120]. Other preparation techniques, like electrospinning [121] and solvothermal synthesis [122], can also be used.

Mesoporous aerogels have a number of applications, including thermal insulation [123,124], electromagnetic wave dispersion [125], CO_2_ capture [126], electrocatalysis (e.g. ethanol oxidation reaction) [127], energy storage system (e.g. supercapacitor) [121], oil refining [128] and adsorption of dyes (both anionic and cationic) [116,129]. Mesoporous aerogels are also desirable for use in therapeutic settings. Mesoporous bioactive glass (MBG) and graphene oxide (GO) combined to form a composite aerogel that demonstrated acceptable hemostatic properties, including low BCI. When MBG–GO is applied to rat liver injury bleeding, it can decrease 60% hemostasis time and reduce 75% blood loss amount compared to medical gauze. Additionally, the composite aerogel exhibits strong photothermal antibacterial properties against *Escherichia coli* and *Staphylococcus aureus* [130].

#### 3.3.3. Macroporous aerogels

The FD technique yields macroporous materials because ice crystals grow during water freezing [131]. They can be employed in different applications such as for sorption-based atmospheric water harvesting (SAWH) [132], drug delivery devices [131] and hemostasis [133]. A three-dimensional macroporous aerogel synthesized from Ti_3_C_2_T_x_ Mxene/cellulose nanofibres/reduced graphene oxide (MCG) where the cellulose nanofibres were used as a bridge to connect the two materials (Mxene and GO). This approach was aiming to solving the challenges of self-stacking between the two-dimensional material surfaces and the tendency of Mxene to oxidize. The three-dimensional MCG aerogel material was employed as a super capacitor and demonstrated an outstanding performance in 5000 cycles with 79.4% retention rate and 60.9 mWh cm^−2^ energy density, particularly at 1.0 mWcm^−2^ power density [134].

### 3.4. Aerogels based on skeletal framework

Aerogels can also be categorized into three groups based on their skeletal structure: (i) aggregates of spheres (e.g. as those in silica aerogels and some polymeric aerogels), (ii) fibrillar networks (e.g. the crystalline strands in syndiotactic polystyrene aerogels or the phase-separated amorphous polymer strands in polyimides polyurea and polydicyclopentadiene), or (iii) aggregates of layered structures (e.g. found in clay aerogels). It is easy to assume that these different skeletal structures offer differences in pore sizes, specific surface area and mechanical properties [55].

### 3.5. Aerogels based on surface chemical properties

Aerogels can be classified as hydrophilic or hydrophobic based on their surface chemical properties [59]. The surface chemical property of an aerogel can be altered by altering the combining ratio of precursors. Hydrophobic silica aerogels can be produced in a variety of ways, including by adding non-polar silica precursors to the sol–gel matrix and altering the matrix’s surface after gelation [135]. For example, a hydrophobic silica aerogel consists of tetramethoxysilane (TMOS) as precursor and methyltrimethoxysilane (MTMS) as a hydrophobic reagent in 1:1.3 ratio, methanol (MeOH) solvent and ammonium hydroxide (NH_4_OH) as a catalyst prepared using the supercritical drying method. The hydrophilic (unmodified) aerogel exhibited water adsorption approximately 4–5 times its own weight within 5 minutes while the hydrophobic (modified) aerogel absorbs <2% water of its own weight for a time period of 1 year [136]. A hydrophobic MTCS-coated loofah sponge (MTCS-LS) was prepared by thermal chemical vapour deposition of methyltrichlorosilane (MTCS) on the loofah sponge. The obtained MTCS-LS aerogel showed selective oil removal from water with a capacity for oil sorption 13 times its dry weight and more than 92% separation efficiency. In another research work, a hydrophilic carbon aerogel for adsorption of Sb (III) was prepared using cerium oxide (CeO_2_) and sulphur. The CeO_2_- loaded and sulphur-doped carbon aerogel (Ce@SCA) demonstrated superior adsorption properties for Sb (III) and high reusability [137].

### 3.6. Aerogels based on precursors (origin)

#### 3.6.1. Inorganic aerogels

Inorganic aerogels with large specific surface area, low density and remarkable mechanical qualities make inorganic aerogels the best candidate materials for application in energy, biomedical, thermal control, catalysis and many others [138]. Conventional inorganic aerogels are often prepared by hydrolyzing and condensing metal alkoxides, e.g. tetraisopropoxyl titanate. A number of semiconductor-based aerogels, such as SiO_2_, ZnO, TiO_2_, InVO_4_, etc., have been used for photocatalytic degradation of different pollutants, including organic dyes, heavy metal ions and gases. Among various inorganic aerogels silica and titania aerogels are the most researched and utilized in the field of adsorption because of their low cost and non-toxicity [31]. Inorganic aerogels can be classified into oxide-based, carbide-based, nitride-based, metal [53] and sulphide aerogels [139].

#### 3.6.2. Organic aerogels

Organic aerogels are advantageous over inorganic aerogels as they are less brittle, more stable, lighter, and have stronger covalent bonds. Organic aerogels have low toxicity, high abundance and porous structure. Because of these important attributes, organic aerogels are widely used in many different sectors, including electrochemistry, catalysis, and removal of a range of pollutants from wastewater and air [31]. Organic aerogels include those based on polysaccharides, phenolics, polyols, proteins and carbons. The lack of readily available biocompatibility in inorganic aerogels necessitates the development of organic aerogels [53].

#### 3.6.3. Hybrid aerogels

In order to combine the necessary qualities and to overcome the drawbacks of pure aerogels, hybrid aerogels are required. In order to control hierarchical porosity networks in the aerogel for improved thermal, optical, electrical and surface characteristics, hybrid aerogels, which are combinations of organic-inorganic [140], organic-organic [141] or inorganic-inorganic [142] are a strategic and synergetic means of material development. Despite the apparent saturation of research on hybrid aerogels, there remains ample opportunity to advance these materials through the investigation of methods to control their porous structure, structural stability and sustainability with improved thermal, optical and surface properties, such as hydrophobicity through surface silylation or phase-change material impregnation [143]. Therefore, hybrid aerogels have been the subject of extensive research in recent decades, which has aided in the development of innovative hybrid materials. Multifunctional hybrid materials have become increasingly attractive as potential options for medicine delivery, energy storage, environmental cleanup and sensing. However, improving the mechanical strength without sacrificing the distinctive characteristics of aerogels is still a difficult task [144].

## 4. Application of aerogels

Aerogels have garnered significant attention in the last few decades because of their distinctive physicochemical qualities, such as low density, large specific surface area and good optical transmittance. Because of these special qualities, aerogels are widely used in wastewater and air treatment [25], solar cells [145,146], thermal insulation [147,148], biomedical [149,150], environmental, energy storage [151,152], catalyst, etc. [31].

### 4.1. Aerogels for water treatment

#### 4.1.1. Polystyrene-graphene aerogel for water treatment

**Figure 4.**
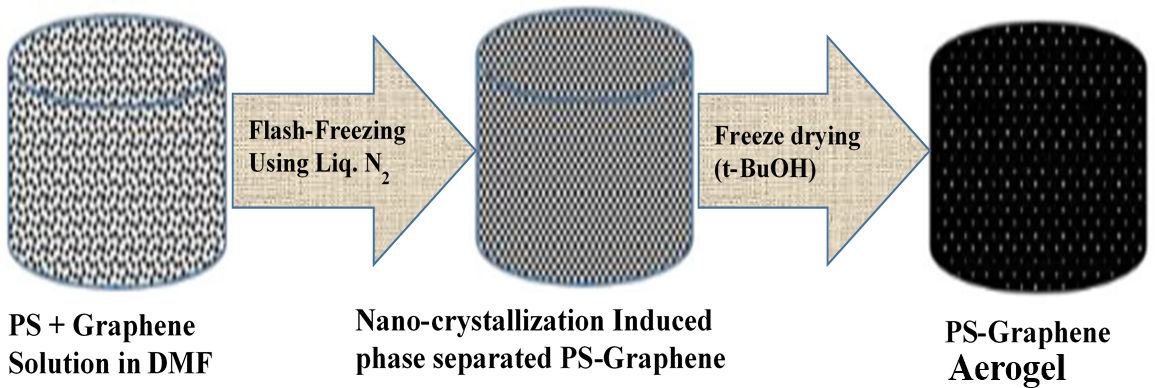
Schematic illustration of PS-G nanocomposite aerogel preparation [152].

Krishnan and Alsharaeh prepared a polystyrene-graphene (PS-G) aerogel using solvent crystallization-induced phase separation through flash freezing route and FD (figure 4). The PS-G nanocomposite aerogel displayed three-dimensional-interpenetrating network architectures with high specific surface area (approx. 300 m^2^/g) and high porosity. The composite aerogel was found to be a potential absorbent for fast removal of oil from the produced water sample. The oil absorption capacity of the PS-G nanocomposite aerogel was 50 g/g, and it reached saturation in 20 min. Moreover, it was discovered that the aerogel absorbent could be effectively recycled up to 10 cycles of washing with ethanol and showed just a slight decrease in oil absorption effectiveness. Additionally, compared to PS aerogel (124 KPa), the PS-G aerogel demonstrated a reinforced compression strength of 152 KPa [153].

#### 4.1.2. Alginate-based aerogels for water treatment

**Figure 5.**
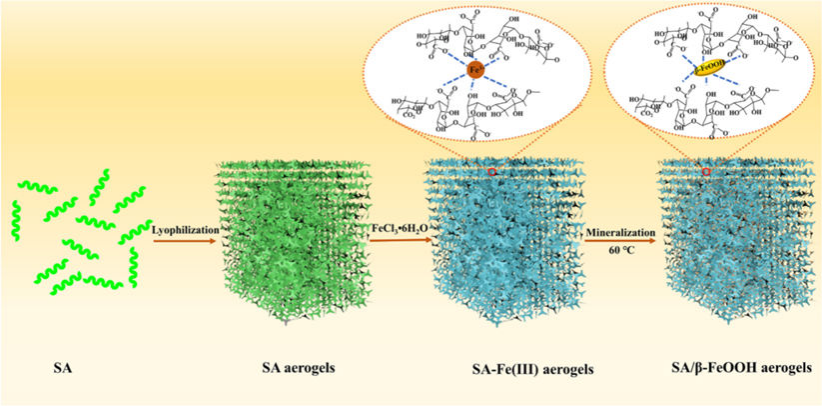
The schematic diagram of preparing SA/β-FeOOH aerogels [153].

In another investigation [154], alginate-based aerogels were prepared for multifunctional water treatment. As illustrated in figure 5, the β-FeOH nanoparticles that are produced on entire alginate aerogels give porous hierarchical topography and additional -OH groups, which enhance underwater oleophobicity and fouling resistance of the porous aerogels. The sodium alginate (SA) aerogels decorated with -FeOH nanoparticles (SA/α-FeOH) were prepared using the chelation and in-situ mineralization methods. The pure SA aerogel that was produced by FD was submerged in a solution containing Fe^+3^ for 96 h with continuous string. Ultimately, the SA/FeOH aerogels were obtained by thoroughly washing them in water and drying them in an oven set at 40°C for 12 h. The porous SA/β-FeOH aerogels demonstrated outstanding water and oil separation selectivity (>99.5%). The aerogels also exhibited Photo-Fenton self-cleaning capabilities for the breakdown of wastewater containing soluble organic contaminants under sunlight.

#### 4.1.3. Nanocrystalline cellulose-based aerogel for water treatment

**Figure 6.**
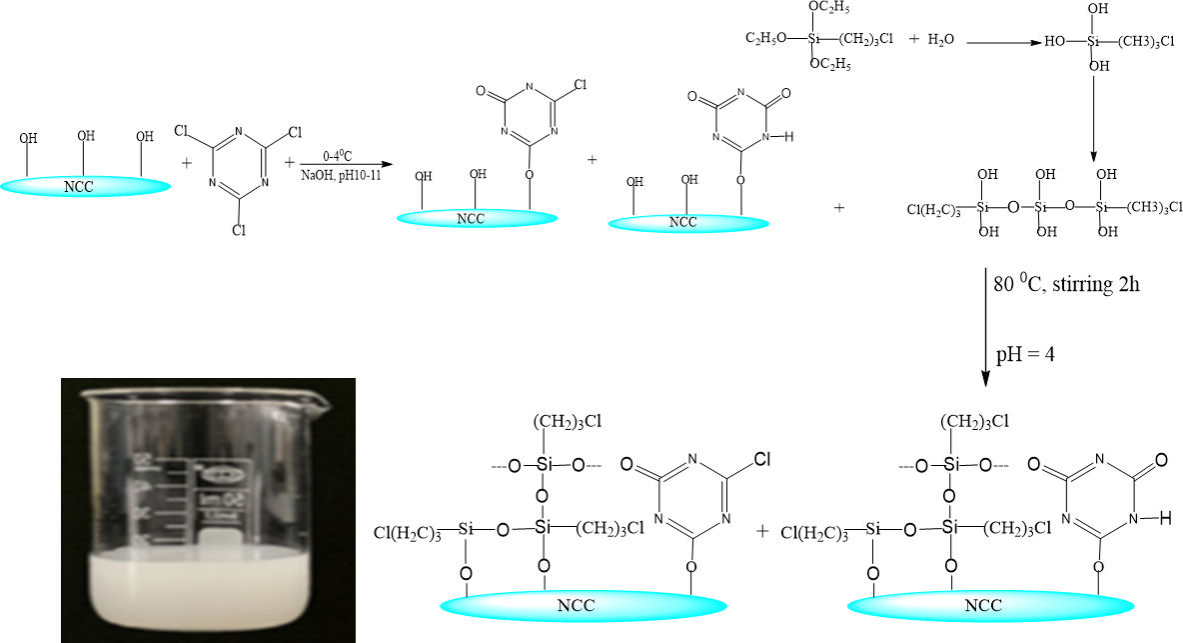
Schematic of the synthesis of NCC-CYCH siloxane hydrogels. The photo in the beaker is the NCC-CYCH siloxane hydrogels [154].

Zhang *et al*. [155] prepared highly porous (>95.2%), hydrophobic and reusable nanocrystalline cellulose (NCC)- based aerogel for water and antibacterial treatment (figure 6). The aerogel was prepared by attaching cyanuric chloride (CYCH) on NCC and using chloropropyltriethoxysilane (CPTES) as a cross-linker. During the test, the NCC-CYCH aerogel (unmodified with silane cross-linker) sank at the bottom of the oil-water mixture (dodecane-water mixture) without any oil absorption. However, the NCC-CYCH siloxane aerogel selectively absorbed dodecane from water and floated on the water surface. The aerogel demonstrated excellent absorption capacity (16 g/g) for ten cycles without any significant change in its shape using toluene as a washing solvent. The study showed increasing the CPTES content decreased the porosity of the siloxane aerogel, which could be ascribed to the progressive decrement of the void volume fractions due to thickening of the cellulosic scaffold. The antibacterial activity of the siloxane aerogel was also tested against Gram-positive *S. aureus* and Gram-negative *E. coli* bacteria. The siloxane aerogel before chlorination showed 1.54 log and 0.80 log reduction of *S. aureus* and *E. coli* within 30 min of contact time, respectively, while after chlorination, the aerogel could cause 5.89 log reduction of S. *aureus* and 6.19 log reduction of *E. coli* within 1 and 5 min of contact time, respectively. Furthermore, the aerogel’s outstanding antibacterial qualities were demonstrated by the ability to kill all bacteria within 5 min.

#### 4.1.4. Graphene-based aerogels for water treatment

Graphene-based aerogels have been demonstrating great promise in the area of water treatment [25,153,156]. Ye *et al*. prepared amphiphilic graphene-based aerogel having affinity from polar water to nonpolar organic liquids [157]. The aerogel was prepared using graphene oxide (GO) and polyvinyl alcohol (PVA) materials, and glutaraldehyde as a cross-linker. The amphiphilicity of the aerogel could be tuned by changing the GO and PVA feeding ratio. The low feeding ratio of PVA to GO produced hydrophobic aerogel, which was used as an adsorbent for organic liquid, while the high feeding ratio of PVA and GO produced hydrophilic aerogel which could be used to remove water-soluble dye in the wastewater. Even after drying in the air or under exceptionally high compression strain, the aerogel returned to its initial volume.

Gorgolis *et al*. synthesized graphene aerogels (GAs) with two different methods, i.e. FD and ambient pressure drying (APD) methods. Their efficiency for adsorption of several water pollutants has been determined. Although the most popular method for obtaining a stable three-dimensional structure from a hydrogel is FD, APD offers less energy consumption and a more facile approach that could potentially be scaled-up. It was discovered that the aerogels' specific surface areas (SSA) were 27 m^2^/g for the FD and 18 m^2^/g for APD. While the GO reduction was confirmed in both material classes, the C/O ratio measured by XPS for the FD samples was nearly twice as high as that of the APD samples. The D band to G band intensity (ID/IG) ratio that quantifies the defects in the lattice and the conjugation disruption was calculated from Raman spectra of the GAs synthesized by both the FD and APD methods. For comparison, the ID/IG ratio was 1.19 for GAs with FD methods and 0.89 for GAs with APD methods. The photo-catalytic potential of both varieties of aerogels was also tested with the MB dye, with the FD method demonstrating much better results. The authors explained the superior photo-catalytic activity demonstrated by the GA with the FD method was due to the higher degree of reduction that has persisted as well as the structure’s doping with phosphorus from the reducing chemical that was utilized. However, for adsorption of toxic organic solvents and oils like formaldehyde, dichloromethane, acetone, ethanol, methanol, pump oil, castor oil and silicone oil the GAs with the APD method demonstrated superior adsorptions than the FD, despite their lower photocatalytic activity [156].

#### 4.1.5. Graphene oxide-based composite aerogel

A three-dimensional composite aerogel synthesized from GO, carboxymethylcellulose aluminum sulphate (GOCAS) was applied for the removal of lead and zinc metals from wastewater. The GOCAS aerogel was synthesized through ice-templating of GO with carboxymethylcellulose and aluminum sulphate as a cross-linking functionalization. The study reported that the hydroxyl and sulphate groups in the aerogel are responsible for the adsorption of the two metals. The aerogel showed 138.7 mg/g adsorption capacity at GOCAS dose of 20 mg, initial concentration 100 mg/l, temperature of 50°C and 45 min contact time for lead and 52.69 mg/g for zinc at 30 mg, 65 mg/l, 45°C and 40 min contact time. Moreover, investigations on regeneration using hydrochloric acid eluent were carried out for a maximum of four adsorption-desorption cycles with success [158].

### 4.2. Aerogels for air purification

Significant amounts of air pollutants produced by human activities and particulate matter (PM) in the air are a major hazard to public health. Novel aerogel materials synthesized from different materials are promising for capture of PM.

#### 4.2.1. Amino functionalized cellulose aerogel for air purification

**Figure 7.**
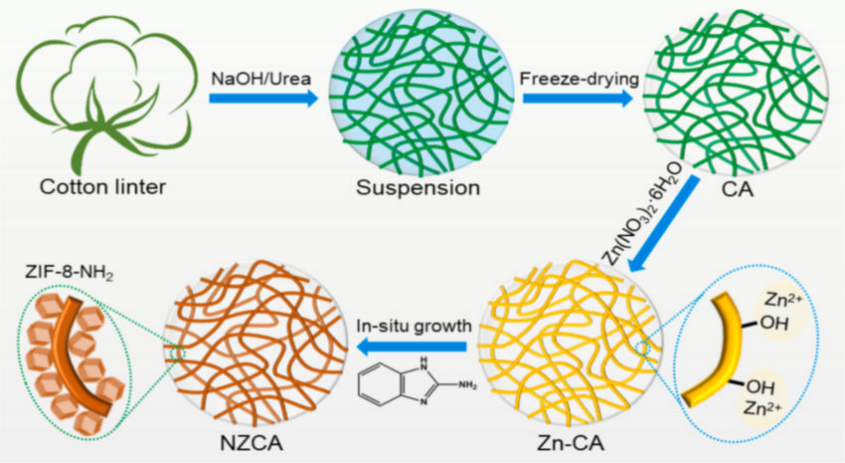
Schematic illustration of the synthesis process of NZCA. Reprinted from an open-access source [158].

Amino (2-aminobenzimidazole) functionalized ZIF−8 was anchored on the surface of cellulose aerogel (CA) to produce NZCA by *in situ* growth approach and tested for air filtration. Figure 7 illustrates how cotton linter was converted into CA by a dissolving, regeneration, FD process in NaOH/urea solvent. This process involves four transformation stages: cotton linter, cellulose suspension, hydrogel and aerogel. Then, CA was used as a substrate for *in situ* growth of ZIF−8-NH_2_ (or ZIF−8) by impregnation in a zinc ion solution. The amino-modified ZIF−8@CA aerogel (NZCA) was obtained after FD and dried. For comparison, ZIF−8@CA (ZCA) can be obtained by changing 2-aminobenzimidazole to 2-methylimidazole. The NZCA demonstrated superior PM filtration performance due to the fact that the functionalized ligand introduces –NH_2_, and the polar –NH_2_ group, enhancing the electrostatic interaction between ZIF−8-NH_2_ and PM. The NZCA performed filtration efficiency and quality factor (PM2.5: 92%, 0.054; PM10: 96%, 0.068) compared to ZCA without sacrificing the pressure drop [159].

#### 4.2.2. A konjac glucomannan-based aerogel for air purification

In another investigation, Qian *et al*. prepared a konjac glucomannan (KGM)-based aerogel for air filtration. As shown in table 2, the KGM-based aerogels were prepared from gelatin, potato starch and wheat straw with different combination ratios of the components. In this investigation, the authors showed how pore-size distribution affects purification time. They demonstrated the purification time could be shortened by over 50% by changing the pore-size distribution from large size to small size or increasing the surface area with the fold structure. The times needed to reduce the PM2.5 concentrations from 500 μg/m3 (heavy air pollution) to <50 μg/m^3^ for K1G2, K1G2S3 and K1G2S3WS1 were 15, 21 13 min, respectively. By adjusting the total solid concentration, the pore-size distribution of the KGM-based aerogel could be controlled. The 50%-K1G2S3, 70% K1G2S3 and K1G2S3 aerogels were used to prepare the flat-structure aerogel air filter with the pore-size distribution from large to small. Thus, the higher the total solid concentration, the smaller the aerogel pore size [160].

**Table 2.** Sample formulae time required to reduce particulate matter (PM) concentrations from 500 μg/m^3^ to *<*50 μg/m^3^.

sample code	KGM (g/100 ml)	gelatin (g/100 ml)	potato starch (g/100 ml)	wheat straw (g/100 ml)	time (min)
K1G2	1.0	2.0	0.0	0.0	15
K1G2S3	1.0	2.0	3.0	0.0	21
K1G2S3WS1	1.0	2.0	3.0	1.0	13
70%-K1G2S3	0.7	1.4	2.1	0.0	30
50%-K1G2S3	0.5	1.0	1.5	0.0	—

#### 4.2.3. A cellulose-silica nanofibre aerogel for air purification

**Figure 8.**
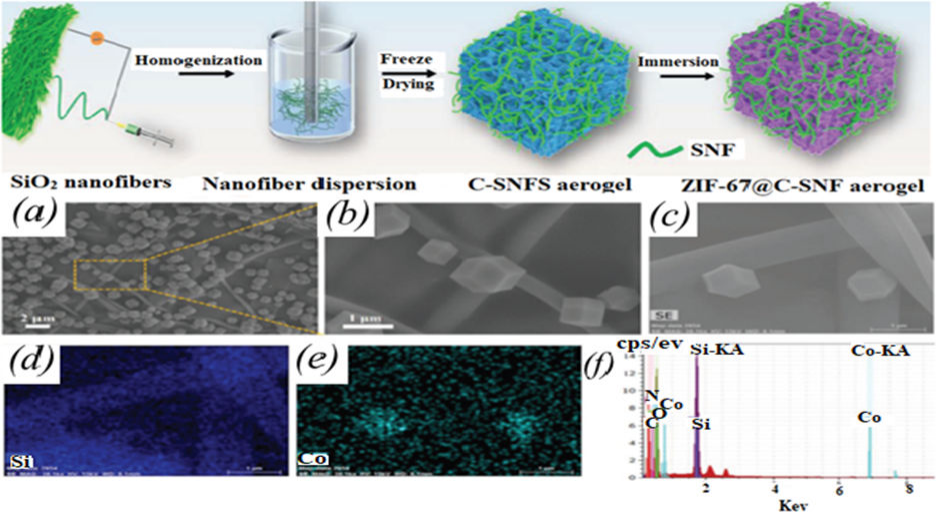
Synthesis routes for ZIF−67@C-SNF. (*a, b*) FE-SEM images of the ZIF−67@C-SNF composite aerogels at different magnifications, (*c*) ZIF−67@C-SNF control image with corresponding elemental mapping images for (*d*) Si and (*e*) Co, (*f*) EDS spectrum of ZIF−67@C-SNF aerogel [160].

A cellulose-silica nanofibre (C-SNF) aerogel loaded with zeolitic imidazolate framework−67 (ZIF−67) was prepared for application in indoor air purification systems using the electrospinning and FD techniques (figure 8). The C-SNF aerogel doped with 0.75 weight percent SiO_2_ nanofibre (SNF) demonstrates a maximum compressive stress of 8.7 MPa, which is higher than the 6.4 MPa of a pure cellulose aerogel. The aerogel retains more than 85% of its original compressive modulus even after 100 compressions. This C-SNF aerogel exhibits a filtration efficiency of 99.91% against 0.3 μm salt particles at 32 l min^−1^air flow rate, which is higher than that of pure cellulose aerogel (51.25%) in similar conditions. Moreover, a consistent removal efficiency of 99.92% for 2.5 μm particulate matter (PM2.5) is observed after 10 cycles. ZIF−67@C-SNF aerogel was prepared by loading a zeolitic imidazolate framework−67 (ZIF−67) onto C-SNF utilizing an *in situ* growing process. The authors clearly demonstrated that the presence of ZIF−67 on C-SNF provides ZIF−67@C-SNF aerogel with high porosity (92.3%,) and renders high removal efficiency (93.75%.) for formaldehyde. The ZIF−67 crystals on the C-SNF aerogel surface were examined using FE-SEM. The high-magnification FE-SEM image clearly shows interpenetrated SNF and rhombic dodecahedral ZIF−67 structures on the walls of the CA, as illustrated in figure 8*a*,*b*. The elemental maps (figure 8*c*–e) of EDS spectral analysis show observations of Si and Co indicating the Si and Co elements are consistently dispersed on the aerogel. Figure 8*f* displays the EDS spectrum with the distinctive C, O, Si, Co and N peaks confirming that ZIF−67 was loaded successfully [161].

### 4.3. Aerogels for supercapacitor

Supercapacitors (SCs) are the emerging energy storage devices that have attracted more and more attention due to the advantages of the rapid charging/discharging rate, ultrahigh power density and super long cycle life. They can be classified into two categories: electrical double layer capacitors (EDLCs) that store energy based on electrostatic accumulation of charges at the electrode/electrolyte interface; and pseudo-capacitors related to the fast and reversible faradic process of the electroactive species within the electrode materials. The capacitances for EDLCs come from carbon materials, such as graphene, porous carbon and carbon aerogel, which are greatly dependent on the surface area of the electrode materials that is accessible to electrolyte ions.

Carbon aerogel is one kind of porous carbon with exceptional three-dimensional network and properties of large specific surface area, chemically inactive and other remarkable physical-mechanical properties, leading to fairly attractive applications in environmental remediation and energy storage and conversion [162]. Graphene, a single layer of hexagonally packed carbon atoms, has long been regarded as an exceptional material for producing SCs because of its mechanical and chemical qualities, high electrical conductivity, stable thermal properties and larger theoretical surface area [163].

#### 4.3.1. Carbon nanofibrous aerogel for supercapacitor

Meso-microporous carbon electrode materials for sustainable and high-performance supercapacitor development by electrospinning polyacrylonitrile (PAN) with F-treated biochar and subsequent aerogel construction is followed by stabilization, carbonization and carbon activation. The resultant carbon nanofibrous aerogel electrode material (ENFA-FBa) exhibited exceptional specific capacitance, attributing to enormously increased micropore and mesopore volumes, much more activated sites to charge storage, and significantly greater electrochemical interaction with electrolyte. This electrode material achieved a specific capacitance of 407 F/g at current density of 0.5 A/g in 1 M H_2_SO_4_ electrolyte, which outperformed the state-of-the-art specific capacitance of biochar-containing electrospun carbon nanofibrous aerogel electrode materials (<300 F/g). A symmetric two-electrode cell with ENFA-FBa as electrode material showed an energy density of 11.2 Wh/kg at 125 W/kg power density. Even after 10 000 cycles of charging–discharging at current density of 10 A/g, the device maintained a consistent coulombic efficiency of 53.5% and an outstanding capacitance retention of 91% [121].

#### 4.3.2. Cellulose carbon aerogel for supercapacitor

**Figure 9.**
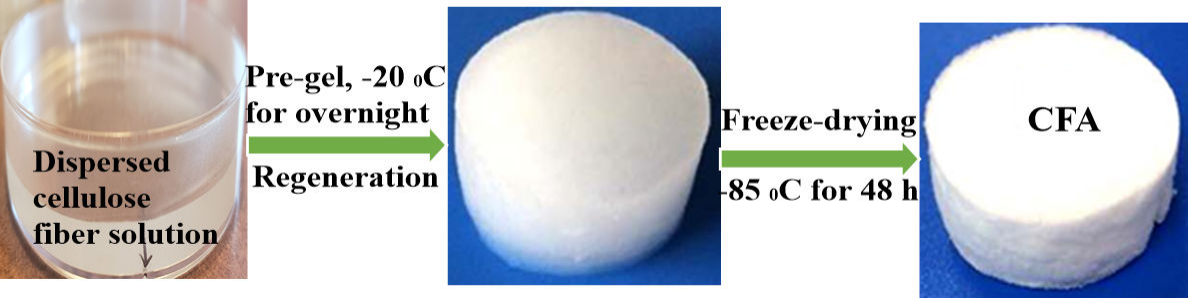
Schematic illustration of the fabrication process of CFA [161].

Yang *et al*. [162] prepared a cellulose carbon aerogel by dissolving bamboo cellulose fibres in an aqueous solution of NaOH and urea. As shown in figure 9, the bamboo fibres were first dissolved in NaOH/urea aqueous solutions with magnetic stirring to obtain a dispersed cellulose fibre sol. Pre-gel was conducted at −20°C overnight followed by immersing into a regeneration bath of ambient DI water. The hydrogel was then cleaned with DI water to remove residual impurities. Subsequently, it was frozen in liquid nitrogen and freeze-dried for 48 h at a temperature of −85°C and a vacuum of 0.06 MPa. It resulted in the formation of cellulose fibre aerogel, which was denoted as carbon fibre aerogel (CFA). The aerogel obtained was applied for supercapacitor. According to the authors, the NaOH/urea solution dissolves the bamboo cellulose fibre and partially breaks down hydrogen bonds in the intra- and inter-cellulose molecular chains enhancing the porosity structure of CFA. This gave the aerogel a three-dimensional network and large specific surface area that is accessible to electrolyte ions. Further research was done to demonstrate how the KOH activation affects the texture and porosity of the cellulose carbon aerogel and how this correlated to its electrochemical performance. Activated carbon aerogel demonstrated a high specific capacitance of 381 F/g in an aqueous electrolyte of 6 M KOH, which was 150% better than the unactivated sample. Because of the suitable micropores and mesopores, a 90% retention rate was obtained at a high scan rate of 200 mV/s.

#### 4.3.3. MXene aerogels for supercapacitor

MXenes are a newly identified class of two-dimensional materials. MXenes' intriguing surface properties and distinct structure have sparked extensive research into their potential applications across numerous fields [164]. However, two-dimensional MXene sheets show significant stacking issues because of hydrogen bonding and van der Waals forces [165–167]. Fast electrolyte ion transport is impeded by this blocking of active sites. It is possible to create MXene pore structures that both prevent interlayer stacking and accelerate ion transport. To greatly increase ion transport efficiency and expose more active sites, new technologies must be developed to design and fabricate three-dimensional hierarchical porous MXene electrode devices with pore architectures of varied scales [164].

**Figure 10.**
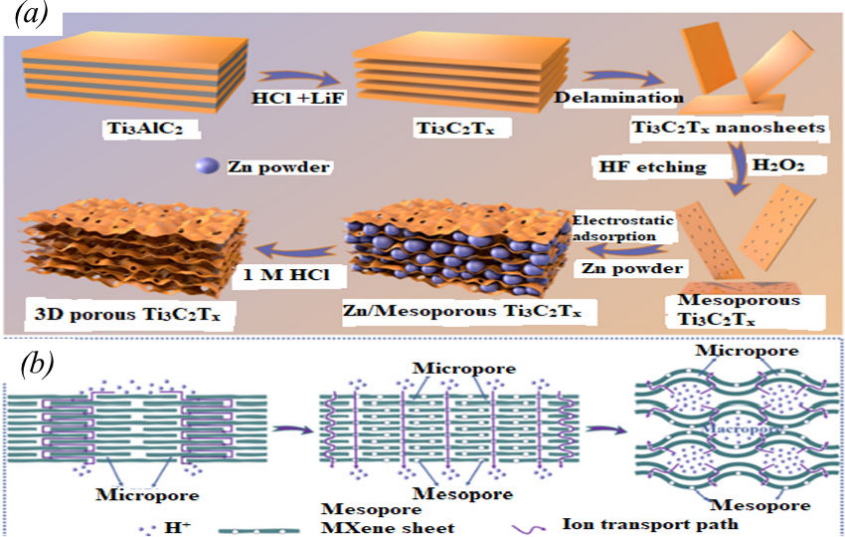
(*a*) Synthesis steps used to obtain the three-dimensional hierarchical porous MXene. (*b*) Schematic diagram of the hydrogen ion diffusion paths through the microporous, mesoporous and macroporous structures [162].

Ji *et al*. [164] prepared MXene-based aerogel with a three-dimensional hierarchical porous structure, which spans the microporous, mesoporous and macroporous scales (figure 10*a*,*b*). According to Ji *et al*., the intercalation of Zn^2+^ into Ti_3_C_2_Tx MXene results in enlarged layer spacing (micropores), H_2_O_2_ oxidation and subsequent acid etching results in the formation of a surface pore structure (mesopores), and the removal of a Zn powder template generates a three-dimensional pore structure (macropores). The constructed three-dimensional hierarchical porous structure exposes more active sites and provides a shorter pathway for electrolyte ion transport. This MXene-based material exhibits significantly improved transport efficiency and electrochemical activity. The Zn-H_2_O_2_-Ti_3_C_2_T_x_ MXene exhibits good capacitance (378.8 F g^-1^ at 2 mV s^−1^ and 391.2 F g^−1^ at 1 A g^−1^) and superb rate performance (retention of 76.7% at 1 V s^−1^). Moreover, this electrode can be stably cycled at 200 mV s 1 (10 000 cycles, retention of 95.88 %). At a power density of 120.8 W kg^−1^, an assembled Zn-H_2_O_2_-Ti_3_C_2_T_x_/carbon cloth flexible symmetric supercapacitor exhibits an excellent energy density of 13.42 Wh kg^−1^.

### 4.4. Aerogels for thermal insulation

#### 4.4.1. Lignocellulose aerogel for thermal insulation

A novel lignocellulose aerogel treated using a recyclable deep eutectic solvent (DES) was physically mixed with tourmaline particles (TPs) to enhance its structural stability, flame retardancy and mechanical properties. The optimized TPs-modified lignocellulose aerogel (TLA−4) had good comprehensive performances due to the synergistic effect of ammonium sulfate and TPs. Compared with TPs-free lignocellulose aerogel (LA), the total heat release (THR) and heat release rate (HRR) of TLA−4 were reduced by 62.0% and 66.3 %, respectively, and the limiting oxygen index (LOI) of TLA−4 was drastically enhanced by 74.1%. TLA−4 also exhibited a low thermal conductivity of 29.67 mW/mK, showing favourable thermal insulation performance. When compressed to 5 %, the mechanical strength of TLA−4 increased by 8.3 times. Meanwhile, the presence of TPs and abundant pores in the aerogel contributed to the release of negative oxygen ions (NOIs), aiding air purification. A life cycle assessment (LCA) indicated that this composite had a minimal environmental impact (EI) in 17 categories compared to other similar aerogels. The proposed strategy for preparing an environmentally-friendly LA offers significant potential for applications in home decoration and building materials [12].

#### 4.4.2. Mullite aerogel for thermal insulation

**Figure 11.**
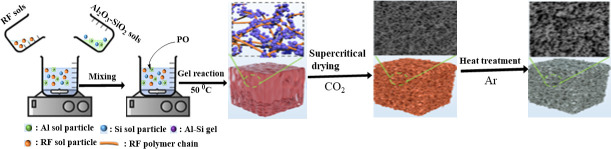
Schematic diagram of the synthesis route of the mullite aerogel. Reprinted from an open-access source [166].

A monolithic mullite aerogel, prepared from resorcinol (R), formaldehyde (F), propylene epoxide (PO) and Al_2_O_3_-SiO_2_ was applied for advanced thermal protection. As shown in figure 11, this RF/ Al_2_O_3_-SiO_2_ aerogel was prepared by the sol–gel method combined with the CO_2_ supercritical drying technique. The mullite aerogel was obtained with the R/Al/Si molar ratio at 1:1:0.5 by heat treatment at 1400 ◦C for 5 h under flowing argon. The resultant mullite aerogel has a large BET specific surface of 481 m^2^/g and a characteristic ‘pearl-necklace’, three-dimensional network with an average pore size of about 10−20 nm. The compressive strength of the mullite aerogel is as high as 15.5 MPa, which is much larger than ceramic aerogels. Excellent thermal insulation properties are displayed by the mullite aerogel, and its rather good anti-oxidation performance is demonstrated by the mass loss of only 2.5% following a 10 minute test using a butane torch at 1300°C under air [168].

#### 4.4.3. Polyurea-cellulose composite aerogel for thermal insulation

Polyurea-cellulose composite aerogel fibres (CAFs) were prepared from a dried bacterial cellulose (BC) and polyurea for thermal insulation. The BC matrix was immersed in a polyurea sol, and the aerogel fibres were obtained via secondary moulding, followed by CO_2_ supercritical drying. The CAFs exhibit outstanding mechanical properties, with a tensile strength of 6.4 MPa. Moreover, the CAFs demonstrate superior thermal insulation capabilities, withstanding temperatures ranging from 180 to −40°C. The SEM images of CAFs show the cross-sections were relatively regular with no evident breakage or shrinkage, which indicated minimal shrinkage during the CO_2_ supercritical drying process. With increasing polyurea precursor concentration, adhesion of polyurea nanoparticles occurred between nanofibres, leading to the gradual formation of lamellar structures. The coating layer of polyurea nanoparticles on nanofibres and the three-dimensional network structure between nanofibres collectively endowed the CAFs with the ability to resist external impact without deformation. However, when the polyurea precursor concentration further increased, a substantial number of polyurea nanoparticles adhered to nanofibre junctions, interweaving to create a gel skeleton and forming a limited number of three-dimensional network structures.

Thermal insulation tests were conducted on cotton thread and silk, both possessing similar diameters and thicknesses to CAFs under identical conditions. When the heat source temperature reached 150°C, the temperature of the CAF mat was approximately 100°C, whereas the surface temperature of the cotton insulation mat and silk insulation mat reached 130 and 135°C, respectively. As the polyurea content in CAFs decreased, the thermal insulation performance gradually deteriorated. This could be ascribed to lower polyurea content resulting in polyurea particles adhering to nanofibres, forming a coating layer without the formation of effective three-dimensional network structures, leading to larger pore sizes between the nanofibres and facilitating convective heat transfer with the surroundings. Therefore, a decrease in the polyurea content diminished the thermal insulation performance of CAFs [169].

### 4.5. Aerogels for drug delivery

Aerogels have become one of the most interesting and promising drug delivery systems due to their extreme adaptability, modularity and industrial manufacturing practicality. They have higher specific surface area and porosity (interconnected mesopores) than other three-dimensional materials. This property may facilitate the loading of small-molecule drugs more quickly, provide less restricted access to the inner regions of the matrix, and improve the interactions between the polymer matrix and the biological milieu. Supercritical CO_2_ medium processing offers notable benefits, including the absence of an oxidizing environment, clean manufacturing and ease of scaling up under excellent manufacturing procedures, for both drug loading (impregnation) and aerogel production (drying) [16].

**Figure 12.**
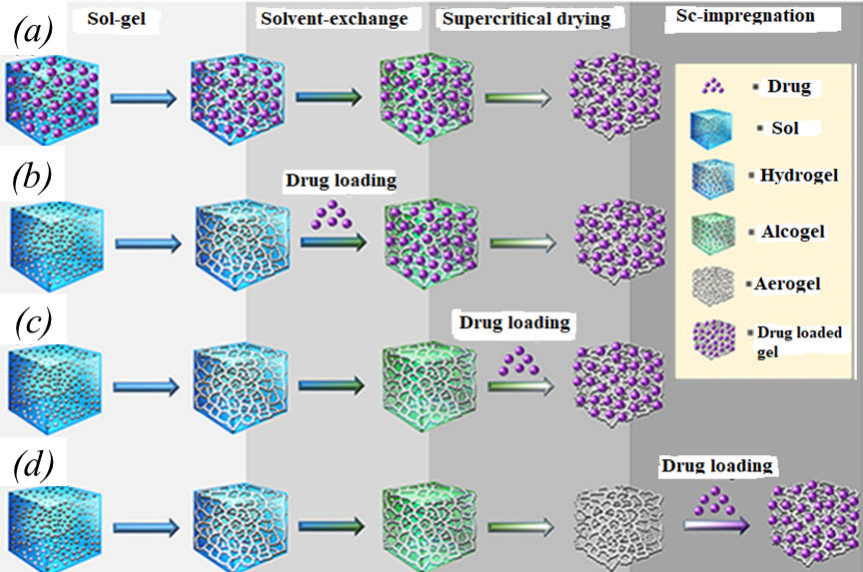
Strategies to load drugs into aerogels: (*a*) the drug is added to the precursors solution; (*b*) the drug is added to the gel; (*c*) the drug is incorporated during the supercritical drying; and (*d*) the aerogel is formed first and then the drug is loaded [15].

Numerous procedures to incorporate drugs either during the manufacturing process or after the aerogels are created have been demonstrated [170]. As demonstrated in figure 12, four strategies to load drugs into aerogels are known and explained as follows:

1. The first and the most straightforward strategy involves incorporating the drug into the gel’s precursor solution (such as polymer dispersion). In order to prevent premature drug extraction, this method uses drugs that are stable under gelling conditions and weakly soluble in the supercritical fluid used for drying as well as in the organic solvents used for solvent exchange [171]. The best example is nicotinic acid which is poorly soluble in water (1.67 g/100 ml), in ethanol (0.7 g/100 ml) and in scCO_2_ (1.84 × 10^–4^ g/ 100 ml, 35°C , 100 bar). Nicotinic acid-loaded alginate aerogels can be made by mixing the medication with the alginate aqueous solution prior to gel crosslinking, ethanol water exchange and scCO_2_ drying. Additionally, the drug’s presence was shown to significantly reduce the alginate hydrogels' shrinkage in ethanol, which promoted the creation of aerogels with larger surface areas. For drugs that are soluble in scCO_2_, water, or organic solvents, FD may be a better processing method than scCO_2_ drying [172].
2. The second method involves first prepping the gel and then immersing it in the drug solution for loading. This method works well for drugs that dissolve in alcohol but not in scCO_2_ [173]. The outcome is the high loading of the drug in the crystalline state. The drug has a limited solubility in water, and its release is primarily controlled by its dissolution, which is enhanced by the aerogel matrix’s greater surface area. For instance, the drug loading process of silica aerogels subjected to a solution of paracetamol is slow. Therefore, in the typical preparation protocol, a gradient of adsorbed drug is generated, i.e. the outer layers of the aerogel (more concentrated) and the inner regions (less concentrated). The soaking time in the drug solution is then short, and the exchange of the solvent by scCO_2_ occurs slowly [174].
3. The drug is incorporated during the supercritical drying. It is possible to load drugs that are soluble in scCO_2_ but sensitive to conventional solvents, such as essential oils or acetylsalicylic acid, during the supercritical drying step of aerogel formation [16].
4. The drug is loaded after the aerogel formation. Drug formulation into preformed aerogels entails exposing the aerogel to drug-containing liquid or gas phases, which must diffuse through the pore network. Depending on its composition, an aerogel may deswell or partially dissolve when submerged in an aqueous or organic solvent. Because of the possibility of pore collapse and the presence of solvent residues that could cause drug instability or toxicity, it necessitates an extra step of solvent removal [16,175].

### 4.6. Aerogels for catalysis

With unique surface characteristics and structural benefits, three-dimensional structures can enhance catalytic activity [127,176,177]. Metallic aerogels (MAs) are special three-dimensional materials having a large specific surface area with self-supporting properties [178,179]. MAs have a three-dimensional crosslinking network structure that promotes mass and electron transport, providing a promising foundation for the development of effective electrocatalysts [180,181].

#### 4.6.1. Metallic aerogels for catalysis

**Figure 13.**
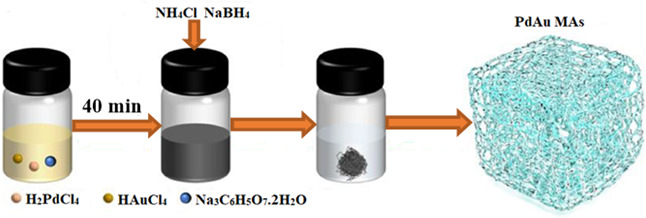
Schematic of Pd_4_Au_3_ MAs synthesis [180].

Dong *et al*. [182] used the liquid-phase reduction approach to successfully prepare three-dimensional network-interwoven PdAu MAs at room temperature (figure 13). To put it briefly, a homogenized mixture solution comprising tetrachloropalladic acid (40 μl of 0.1 M H_2_PdCl_4_), chloroauric acid (30 μl of 0.1 M HAuCl_4_) and trisodium citrate dihydrate (100 μl of 1 M Na_3_C_6_H_5_O_7_⋅2H_2_O) was mixed with sodium borohydride (100 μl 1 M NaBH_4_) as a reducing agent and ammonium chloride (2.5 ml 2 M NH_4_Cl) solutions. A black solution was obtained which was left to stand at room temperature overnight. The sample was freeze-dried for 7 h. The temperature of the cold trap was set at −70°C. Three-dimensional network-interwoven PdAu Mas were obtained. The cross-linking nature of the three-dimensional network facilitated mass transfer and offered a large number of active sites. When compared to Pd MAs/C, commercial Pt/C and Pd/C, the oxygen reduction reaction (ORR), ethylene glycol oxidation reaction (EGOR) and ethanol oxidation reaction (EOR) performances of Pd_4_Au_3_ MAs/C in alkaline medium were much improved and showed exceptional stability. The d-band centre of Pd was modified by the lattice stretching and electronic synergistic effect of the Pd–Au sites, which also improved the adsorption energy of intermediates and caused the cleavage of C–C bonds in ethanol and ethylene glycol. The mass activities of Pd_4_Au_3_ MAs/C in ORR, EGOR and EOR were 1.74, 8.57 and 9.54 A mg_Pd_^−1^, respectively, which were remarkably better than those of referenced Pd MAs/C, commercial Pt/C and Pd/C. The authors explained that the introduction of Au accelerated the kinetics of Pd_4_Au_3_ MAs towards ORR, EGOR and EOR in alkaline electrolyte, boosting the activity and stability of Pd_4_Au_3_ MAs.

## 5. Advanced proposed designs

**Figure 14.**
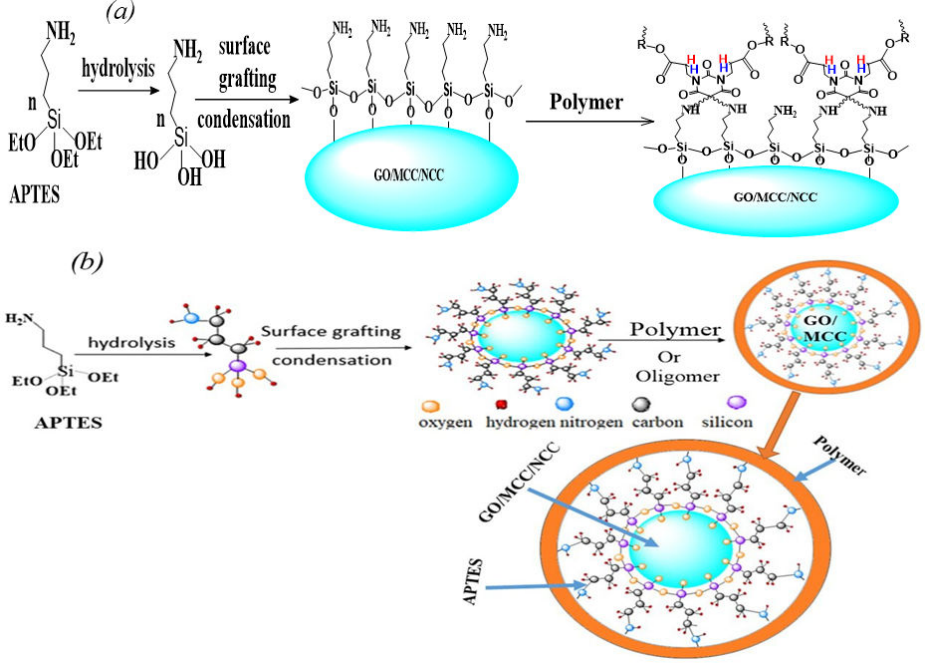
Advanced proposed design for synthesis of aerogel with grafting of (*a*) APTES on GO/MCC/NCC and (*b*) polymer/oligomer coating after grafting of APTES.

Knowing that the design for fabrication of an aerogel has a significant effect on its porosity, specific surface area, density and microstructure and hence on its application, we have proposed the following design (figure 14). In this proposed method, ethoxy silane, like 3-amino propyl triethoxysilane (APTES), can undergo hydrolysis in the presence of solvents such as water, ethanol or other protic solvents. Substrates having -OH moieties such as GO, microcrystalline cellulose (MCC), and NCC can be functionalized through surface grafting and condensation process with APTES. The APTES molecules form a cross-linking structure [183] among themselves through siloxane moieties which reinforce the aerogel structure, strengthening the physical structure. Moreover, the amino group of the APTES can undergo further reactions with vinyl group-containing polymers or oligomers such as bisphenol a diglycidyl ether diacrylate [184]. Such aerogels are hypothesized to have high porosity and extremely large surface area which can be suitable for different applications, mainly for removal of organic and inorganic pollutants from air and water solution. The cross-linking structure not only reinforces the physical structure of the aerogel; it will create numerous micro-channels for the rapid movement of a solution containing pollutants. This aerogel can also be used for thermal insulation due to the fact that the siloxane may eventually be transformed into crystalline SiC and amorphous SiO_2_ particles, which are then deposited on the surface of carbon nanoparticles to increase the aerogels’ resistance to oxidation [185].

## 6. Conclusions

Nowadays, the modern world faces significant challenges in dealing with water and air pollution because of the fast growth of heavy industry and climate deterioration. The effects of some pollutants, such as heavy metal ions, organic dyes, carbon dioxide, nitrogen oxides (NO_x_) and VOCs, on the environment have come under close examination in recent years. These pollutants may be poisonous or cancer-causing, which could have negative consequences on the environment as well as human health. Therefore, developing an economical and successful strategy to remove these toxins from the environment is of great interest. Compared with conventional adsorbents, nanostructures, etc., aerogels prepared from organic, inorganic and hybrid materials showed fascinating performance in different applications. Aerogels are highly porous, light in weight and have an extremely high surface area. Because of their outstanding physical and chemical properties with adjustable surface chemistry, aerogels have been widely applied to remove pollutants, such as heavy metal ions, dyes, PAHs, oils and organic solvents. In addition, aerogels have various other applications such as biomedical devices, catalysts, drug delivery, food technology and thermal insulation, chemical sensors, energy storage, acoustic transducers, waterproof coatings, wearable fabrics and smart devices to mention a few. The applications and promising prospects of aerogels in environmental pollution management and many other applications are summarized. Furthermore, the interaction mechanisms between pollutants and the porous materials are well explained. More importantly, knowing the design for the fabrication of an aerogel has a significant effect on its porosity, specific surface area, density and microstructure, new designs have been proposed that will be very helpful for researchers in the area.

## Data Availability

This article has no additional data.
